# Prevalence, Awareness, Treatment, and Control of Hypertension in an Isfahan State Institution Sample

**Published:** 2018-04

**Authors:** Bahram Pakzad, Mojtaba Akbari, Fatemeh Baberi

**Affiliations:** 1 *Internal Medicine Department, School of Medicine, Isfahan University of Medical Sciences, Isfahan, Iran.*; 2 *Department of Epidemiology, School of Health, Shiraz University of Medical Sciences, Shiraz, Iran. *; 3 *Department of Occupational Health, School of Health, Shiraz University of Medical Sciences, Shiraz, Iran.*

**Keywords:** *Hypertension*, *Prevalence*, *Awareness*, *Therapeutics*, *Social class*

## Abstract

**Background:** Hypertension is a major risk factor for premature disability and death and is the leading risk factor for global disease burden. The present study aimed to assess the rates of prevalence, awareness, treatment, and control of hypertension in a sample of teachers, staff, and students at Isfahan University of Medical Sciences (IUMS).

**Methods:** This cross-sectional survey was conducted from January to September 2015 on the staff, teachers, and students at IUMS. A total of 1500 subjects were randomly selected and were invited to participate in the survey. Hypertension was defined as an average of 2 blood pressure measurements of at least 140/90 mmHg. The rate of awareness was determined based on self-reports, treatment was defined as the regular use of blood pressure-lowering medications, and control was defined as the maintenance of blood pressure below 140/90 mmHg.

**Results: **The study population comprised 1317 individuals (45.9% female) at a mean age of 41.4±9.5 years. The prevalence rate of hypertension was 17.5% (231 of 1317 participants), and the rate of awareness was 54.5% (126 of 231 hypertensive patients). Seventy-nine (62.7%) patients were undergoing treatment, and 51.9% (41 of 79) controlled their disease. Institutional position (P<0.017), age (P<0.001), body mass index (P<0.001), education level (P<0.001), smoking status (P<0.001), and history of diabetes mellitus (P<0.001) were the most frequent risk factors associated with hypertension.

**Conclusion:** The percentage of the hypertensive subjects who were aware, treated, and controlled was unacceptably low in our sample at IUMS. Hypertension is, therefore, a major health problem in this state institution.

## Introduction

Worldwide, hypertension is one of the most common chronic diseases. It is deemed not only a major risk factor for premature disability and death but also the leading risk factor for global disease burden.^1^ In Western and Asian populations, hypertension is the major cause of stroke, congestive heart failure, and other cardiovascular diseases (CVDs)-themselves the leading causes of morbidity and mortality. Cardiovascular morbidity and mortality can be meaningfully diminished after a reduction in blood pressure (BP).^2^ The past decade has witnessed rapid economic development In Iran and its resultant impact on lifestyle and diet, both of which may have exerted a great impact on the prevalence and incidence of atherosclerotic diseases. A 25% relative reduction in the prevalence of hypertension in the time period between 2015 and 2025 is one of the objectives in the action plan of the World Health Organization for the prevention and control of noncommunicable diseases (Iso H, Kokubo Y. Global strategies for non-communicable disease in terms of predictive, preventive and personalized medicine. EPMA J 2014;5:A4).

In different studies carried out in the Mediterranean and Middle Eastern countries, the prevalence rate of hypertension has been reported to range from 10% to 17%.^3^ In general, 25% to 35% of middle-aged Iranians are hypertensive.^4^ Based on previous studies in Iran, the prevalence of hypertension did not change between 2000 and 2007, whereas there was an increase in the control of hypertension among individuals with hypertension.^5^ A study demonstrated that despite significant improvement in awareness, treatment, and control of hypertension among hypertensive individuals, only half of them were aware of their high BP, half of these aware patients received treatment, and almost half of these treated patients had an adequately controlled BP.^6^^, ^^7^ In a nationwide survey, the prevalence of hypertension in the Iranian adult population (aged 25-64 y) was 25%; nonetheless, only 34% of these hypertensive patients were aware, 25% were taking antihypertensive medications and of these treated subjects, only 24% controlled their hypertension.^8^ Other studies have reported different values apropos the prevalence, awareness, treatment, and control of hypertension.^9^^-^^13^

In light of the aforementioned evidence, underscoring the undesirable status of awareness, treatment, and control of hypertension despite recent improvements in Iran, we sought to perform the present study on a small sample of teachers, students, and staff at an Iranian state institution (Isfahan University of Medical Sciences [IUMS]) in order to assess the prevalence, awareness, treatment, and control of hypertension.

## Methods

This cross-sectional survey was conducted from January to September 2015 on teachers, students, and staff at IUMS, Isfahan, Iran. A total of 1500 subjects were randomly selected and were invited to participate in the survey. Of these subjects, 183 individuals were excluded due to incomplete data and unwillingness to participate. The survey was completed by 1317 subjects. The inclusion criteria were comprised of current employment or education at IUMS, provision of verbal consent for participation, minimum age of 20 years (both males and females), and mental competence. The exclusion criteria consisted of pregnancy, history of heart attack or stroke, and surgery in the preceding 3 months. The study protocol was approved by the Ethics Committee of IUMS.

The questionnaire used in the present study contained information about age, gender, height, weight, education level, marital status, self-reported smoking status, history of diabetes mellitus (DM), family history of hypertension and CVDs, and BP measurement. All the measurements were taken by 2 specially trained nurses. The participants were stratified in 3 age groups: 20 to 39, 40 to 59, and 60 years of age and older. The body mass index (BMI) was estimated through the division of the body weight (kg) by the square of the body height (m^2^) and was categorized as less than 25, between 25 and 29, and equal to or greater than 30 kg/m^2^. Education level was classified as high school diploma and lower, bachelor’s degree, master’s degree or MD, and PhD or specialty and higher degrees. Marital status was categorized as married, divorced, widowed, and never married.

Blood pressure was measured on the left arm of all the respondents after a rest of 5 minutes in the sitting position at the same time of the day, using a standard sphygmomanometer and cuffs of appropriate sizes. Two consecutive BP measurements were taken with at least a 5-minute interval between them, and the average of these 2 measurements was factored in the analysis. Hypertension was defined as an average systolic BP (SBP) of at least 140 mmHg or an average diastolic BP (DBP) of at least 90 mmHg, or if the participant was already taking antihypertensive medications.^14^

The rates of hypertension prevalence, awareness, treatment, and control were evaluated in the participants.^15^^-^^18^ Awareness of being hypertensive was defined as a “Yes” answer to the questionnaire’s item “Have you ever been told by a doctor or a health professional that you had hypertension, also called 'high blood pressure'?” The respondents who were aware of their hypertension were to answer the next item on the questionnaire “Because of your high blood pressure, are you now taking prescribed medicines?” A “Yes” answer was designated as treatment of hypertension. Additionally, in those taking medications for hypertension, an average SBP below 140 mmHg and an average DBP below 90 mmHg hypertension constituted hypertension control. Among the individuals with DM, an average SBP below 130 mmHg and an average DBP below 85 mmHg were taken to represent hypertension control. 

All the statistical analyses were done using the SPSS software package for Windows, version 23 (SPSS Inc., Chicago, USA). The continuous variables are presented as means±standard deviations (SDs) and the qualitative variables as numbers (percentages). The χ^2 ^test with the Yates correction, where appropriate, was employed to compare the frequencies. A multivariate logistic regression analysis was also conducted to evaluate the association between the prevalence of hypertension and age (20-39=1, 40–59=2, and ≥60=3), sex (male=0 and female=1), BMI (<25 kg/m^2 ^=1, 24–29 kg/m^2^=2, and ≥30 kg/m^2 ^=3), educational attainment (high school diploma and lower=1, bachelor’s degree=2, master’s degree or MD=3, and PhD or specialty and higher degrees=4), marital status (married=1, divorced=2, widowed=3, and never married=4), and smoking status (nonsmokers=0 and smokers=1). All the probability tests used in the current study were 2-tailed and the alpha was set at 5%.

## Results

The response rate in our study was 87.8% (1317 of 1500 subjects). The mean age of the subjects was 41.4±9.5 years. The characteristics of the participants are depicted in Table 1. Those aged between 35 and 49 years accounted for the highest proportion of the study population. Generally, the level of education among the respondents was high, with the majority holding a bachelor’s degree. However, 5.8% of the participants had no formal education. The university staff comprised the bulk of the study population (48.7%), and only a small number of the respondents were teachers with PhD or specialty and higher degrees. The overall prevalence rate of overweight and obesity was 30.9% and 7.9%, correspondingly. Most of the subjects (68.2%) were married. The proportion of smokers was 18.1%, and 11.3% of the participants suffered from DM.

The prevalence of hypertension in the whole study population was 17.5% (231 of 1317 subjects). Of these hypertensive individuals, 54.5% (126 of 231) were aware of their hypertension; 62.7% (79 of 126) of the subjects who were aware of their hypertension were taking antihypertensive drug treatment; and 51.9% (41 of 79) of those undergoing treatment had hypertension control. The prevalence of hypertension and the level of awareness, treatment, and control of hypertension by sociodemographic characteristics in the studied subjects are shown in Table 2. As is shown in this table, the prevalence of hypertension in the university staff was significantly higher than that in the teachers or students and the students were more aware of their hypertension. Nonetheless, there were no significant differences between the staff, teachers, and students in the rate of treatment and control of hypertension. The prevalence of hypertension increased with age, with the studied subjects more than 50 years old having a significantly higher prevalence of hypertension than the younger subjects. The prevalence of hypertension significantly increased with a higher BMI and significantly decreased with a higher education level. The divorced and widowed individuals had a significantly higher prevalence of hypertension than the married and single subjects. The diabetic subjects had a significantly higher prevalence rate of hypertension than those without DM (40.3% vs. 14.4%, respectively). Smoking and family history of hypertension and CVDs were significantly associated with higher prevalence rates of hypertension. The level of awareness was associated with the BMI, institutional position, smoking, and family history of hypertension and CVDs. The overweight and obese subjects were more aware of their hypertensive status than those with a normal BMI. History of DM was the only significant variable associated with the rate of treatment, and the level of hypertension treatment was not significantly different between the respondents in regard to the other studied variables. The rate of controlled hypertension was not significantly different between the participants concerning any of the studied variables. 

The odds ratios of the factors potentially associated with the prevalence of hypertension and the level of awareness, treatment, and control of hypertension in the study population are presented in Table 3. Our logistic regression analysis revealed that institutional position, age, BMI, educational attainment, smoking status, and history of DM comprised the risk factors most frequently associated with a diagnosis of hypertension. The subjects younger than 35 years or between 35 and 50 years were less likely than those older than 50 years to be hypertensive (OR: 7.3 [95%CI: 3.95–13.48] and OR: 5.1 [95%CI: 2.42–10.33], respectively). The overweight subjects or the normal-weight subjects were less likely than the obese subjects to be hypertensive. A higher level of education was shown to be associated with potential protection against the incidence of hypertension. Among the hypertensive subjects, the smokers and those with a family history of hypertension were more aware of having hypertension than the others (OR: 3.1 [95%CI: 1.20–7.84] and OR: 4.5 [95%CI: 2.31–8.65], correspondingly); among the subjects aware of their hypertension, a minimum of 16 years of education was significantly associated with a higher likelihood of receiving pharmacological treatment for hypertension (OR: 0.2 [95%CI: 0.04-0.97]); and among those treated with antihypertensive drugs, being divorced and widowed was the only significant variable allied to hypertension control (OR: 3.9 [95%CI: 1.05–18.10]).

**Table 1:** Characteristics of the study participants (N=1317)*

Gender	
Female	604 (45.9)
Male	713 (54.1)
Age groups (y)	
20–34	333 (25.3)
35–49	685 (52.0)
≥50	299 (22.7)
Body mass index (kg/m^2^)	
Normal (<25)	806 (61.2)
Overweight (25–29)	407 (30.9)
Obesity (≥30)	104 (7.9)
Institutional position	
Teacher	214 (16.3)
Staff	642 (48.7)
Student	461 (35.0)
Education attainment (y)	
<7 (primary school)	76 (5.8)
7–12 (high school)	187 (14.2)
13–16 (bachelor’s degree)	541 (41.1)
17–18 (master’s degree or MD)	301 (22.9)
>17 (PhD or medical specialty)	212 (16.4)
Marital status	
Married	898 (68.2)
Divorced/Widowed	73 (5.5)
Never married	346 (26.3)
Smoking	238 (18.1)
History of diabetes mellitus	149 (11.3)
Family history of hypertension	486 (36.9)
Family history of cardiovascular diseases	137 (10.4)

* Data are presented as n (%)

**Table 2:** Prevalence, awareness, treatment, and control of hypertension by sociodemographic factors in the study population (N=1317)*

	Hypertension (n=231)	Awareness (n=126)	Treated (n=79)	Controlled (n=41)
Institutional position				
Teacher	18 (8.4)	5 (27.8)	5 (100)	3 (60.0)
Student	71 (15.4)	45 (63.4)	30 (66.7)	15 (50.0)
Staff	142 (22.1)	76 (53.5)	44 (57.9)	23 (52.3)
P	< 0.001	0.024	0.134	0.915
Age groups (y)				
20–34	37 (11.1)	19 (51.4)	14 (73.7)	8 (57.1)
35–49	75 (10.9)	47 (60.3)	28 (59.6)	14 (50.0)
≥50	119 (39.8)	60 (50.4)	37 (61.7)	19 (51.4)
P	< 0.001	0.227	0.548	0.905
Gender				
Female	129 (21.4)	62 (48.1)	37 (59.7)	19 (51.4)
Male	108 (15.1)	64 (59.3)	42 (65.6)	22 (52.4)
P	0.921	0.072	0.455	0.927
				
Body mass index (kg/m^2^)				
<25	78 (9.7)	32 (41.0)	19 (59.4)	11 (57.9)
25–29	73 (17.9)	45 (61.6)	24 (53.3)	14 (58.3)
≥30	80 (76.9)	49 (61.3)	36 (73.5)	16 (44.4)
P	<0.001	0.013	0.118	0.479
Family history of CVD	46 (33.6)	31 (67.4)	21 (67.7)	12 (57.1)
P	<0.001	0.011	0.895	0.575
Educational attainment (y)				
<7	50 (65.8)	23 (46.0)	16 (69.6)	7 (43.8)
7–12	87 (46.5)	49 (56.3)	28 (57.1)	12 (42.9)
12–16	61 (11.3)	39 (63.9)	27 (69.2)	16 (59.3)
16–18	25 (8.3)	12 (48.0)	6 (50.0)	4 (66.7)
>18	8 (3.8)	3 (37.5)	2 (66.7)	2 (100)
P	<0.001	0.275	0.615	0.549
				
Marital status				
Married	146 (16.3)	78 (53.4)	48 (61.5)	28 (58.3)
Never married	56 (16.2)	32 (57.1)	18 (56.3)	8 (44.4)
Divorced/Widowed	29 (39.7)	16 (55.2)	13 (81.3)	5 (38.5)
P	<0.001	0.891	0.227	0.343
Smoking	179 (75.2)	101 (56.4)	63 (62.4)	35 (55.6)
P	<0.001	0.077	0.881	0.197
History of DM	60 (40.3)	30 (50.0)	16 (53.3)	6 (37.5)
P	<0.001	0.884	0.049	0.197
Family history of HTN	144 (29.6)	95 (66.0)	65 (68.4)	32 (49.2)
P	<0.001	<0.001	0.439	0.306

DM, Diabetes mellitus; HTN, Hypertension; CVD, Cardiovascular disease

* Data are presented as n (%).

**Table 3:** Factors associated with the prevalence, awareness, treatment, and control of hypertension

	Hypertension		Awareness		Treated		Controlled	
	OR (95%CI)	P	OR (95%CI)	P	OR (95%CI)	P	OR (95%CI)	P
Institutional position								
Teacher	Reference	0.017		0.393		0.882		0.275
Student	0.2 (0.04-0.78)	0.006	2.4 (0.55-10.42)	0.238	1.3 (0.88-1.93)	0.634	0.6 (0.16-2.08)	0.366
Staff	1.2 (0.63-2.30)	0.918	1.9 (0.79-4.49)	0.266	0.9 (0.28-2.20)	0.675	0.5 (0.11-1.74)	0.117
Age groups (y)								
20–34	Reference	<0.001		0.363		0.416		0.831
35–49	5.1 (2.42-10.33)	<0.001	0.9 (0.38-2.26)	0.965	0.7 (0.19-2.73)	0.642	0.8 (0.17-3.88)	0.792
≥50	7.3 (3.95-13.48)	<0.001	0.7 (0.34-1.23)	0.168	1.6 (0.64-4.24)	0.319	1.3 (0.39-4.13)	0.981
Gender (Female)	1.1 (0.67-1.87)	0.870	0.6 (0.34-1.12)	0.111	1.0 (0.41-2.36)	0.932	1.1 (0.36-3.15)	0.875
BMI (kg/m^2^)								
<25	Reference	<0.001		0.070		0.310		0.222
25–29	12.1 (4.91-29.23)	<0.001	1.9 (0.85-4.36)	0.061	1.3 (0.37-4.55)	0.716	0.3 (0.05-2.10)	0.164
≥30	10.8 (4.33-27.58)	<0.001	0.7 (0.34-1.53)	0.632	2.1 (0.82-5.50)	0.120	0.9 (0.24-3.24)	0.674
Educational attainment (y)								
<7	Reference	<0.001		0.340		0.178		0.081
7–12	0.1 (0.01-0.39)	<0.001	1.7 (0.27-11.25)	0.681	0.2 (0.05-1.21)	0.291	0.6 (0.11-44)	0.432
12–16	0.1 (0.03-0.79)	<0.001	0.9 (0.12-5.82)	0.853	0.5 (0.12-2.02)	0.489	3.1 (0.49-19.78)	0.545
16–18	0.1 (0.00-0.24)	<0.001	0.7 (0.08-4.83)	0.631	0.2 (0.04-0.97)	0.021	0.6 (0.22-1.36)	0.361
>18	0.4 (0.08-2.07)	0.134	2.2 (0.31-16.36)	0.688	0.3 (0.02-0.87)	0.011	0.3 (0.06-1.82)	0.152
Marital status								
Single	Reference	0.004		0.483		0.137		0.116
Married	0.2 (0.08-0.55)	<0.001	0.7 (0.32-1.37)	0.266	1.5 (0.56-4.27)	0.392	1.9 (0.52-7.02)	0.392
Widowed/Divorced	0.8 (0.42-1.45)	0.572	1.2 (0.47-3.19)	0.822	0.3 (0.06-1.22)	0.078	3.9 (1.05-18.10)	0.044
Smoking	49.9 (26.08-95.67)	<0.001	3.1 (1.20-7.84)	0.008	0.9 (0.23-3.16)	0.894	7.7 (1.14-52.29)	0.069
History of DM	4.6 (2.07-9.89)	<0.001	0.8 (0.42-1.78)	0.899	0.3 (0.11-0.86)	0.023	0.3 (0.07-1.75)	0.239
Family history of HTN	3.3 (1.80-6.11)	<0.001	4.5 (2.31-8.65)	<0.001	1.9 (0.65-5.43)	0.239	0.4 (0.09-1.83)	0.153
Family history of CVD	1.1 (0.51-2.53)	0.414	1.8 (0.81-3.91)	0.103	0.6 (0.23-1.65)	0.340	1.3 (0.33-5.01)	0.449

BMI, Body mass index; DM, Diabetes mellitus; HTN, Hypertension; CVD, Cardiovascular disease

## Discussion

In Iran, a great many people are state-employed and it seems necessary that the prevalence of hypertension in this population be determined and compared with that in the general population. We conducted the present study on a large representative sample at a state institution using standard protocols and instruments to explore the prevalence, awareness, treatment, and control of hypertension in this population. We found that the prevalence rate of hypertension among the staff, teachers, and students at IUMS was 17.5%. Of these hypertensive individuals, 54.5% were aware of their hypertension, more than half of the aware subjects were taking antihypertensive drug treatment, and almost half of these on-drug patients had an adequately controlled BP. A higher prevalence rate of hypertension was significantly associated with institutional position, older age, higher BMI, lower educational attainment, smoking, and positive history of DM. Obesity and family history of hypertension were significant factors allied to awareness of having hypertension. Institutional position was the only significant factor linked to receiving pharmacological treatment for hypertension. Our findings showed that the prevalence, awareness, treatment, and control of hypertension in our study subjects, who are in frequent contact with health-care and information centers, were far from satisfactory. In our study, with the exception of age, the other variables associated with hypertension are modifiable. Given that the population pyramid in Iran predicts the aging of the general population in the near future, policy makers should pay sufficient heed to hypertension control. 

In a cross-sectional study with a large sample size, aged 35 to 70 years, from 628 communities in 17 countries with different income levels, Chow et al.^19^ reported that 40.8% of all the participants had hypertension. Of these hypertensive patients, 46.5% were aware of the diagnosis; 87.5% of those who were aware of their diagnosis were receiving pharmacological treatment; and 32.5% of the on-drug individuals had hypertension control. The authors also showed that the level of awareness, treatment, and control of hypertension was lower in communities with lower incomes. In the years 1999 to 2004, the overall prevalence of hypertension among American adults was 29.3%.^20^ The overall prevalence of hypertension was reported to be 27.9% in the Chinese general population^21^ and 30.0% among some Chinese state institutional employees.^22^ In a survey in Canada, 82% of the individuals who were aware of their hypertension received treatment.^23^ In the China National Nutrition and Health Survey of 2002, the rate of awareness of hypertension was 28% with a treatment rate of 78%.^24^ In a survey in the United States among all the studied subjects with hypertension, 82% were aware, 79% were on treatment, and 39% controlled their hypertension.^25^ In a study in India, 42.8% of the studied women were aware of their hypertension and the treatment rate was 38.6%.^26^ Authors in the first nationally representative study in Iran reported that 25% of adults aged between 25 and 64 years had hypertension, 34% were aware of their diagnosis, 25% were taking prescribed medication, and only almost 6% controlled their hypertension.^8^ Another national study reported that 40.5% of people with a low socioeconomic status in Iran had hypertension, in contrast to 16.4% of hypertensive patients with a high socioeconomic status.^27^ In some other cities of Iran, the prevalence of hypertension is reported to range between 17% and 22%, with different rates of awareness, treatment, and control of hypertension.^5^^-^^8^ Shirani et al.^8^ in their study on the general population of Isfahan reported that the rate of prevalence, awareness, treatment, and control of hypertension was 17.3%, 40.3%, 35.3%, and 9.1%, respectively. In the present study, the prevalence rate of hypertension among the staff, teachers, and students at IUMS was 17.5%, which was lower than most of the rates in other studies both in other Iranian cities and in other countries. The discrepancies between the findings can be explained by the different samples analyzed in the investigations. Whereas the studied subjects in our investigation were state-employed with relatively easy access to the health-care system, the other relevant studies assessed hypertension in general populations. The rate of awareness in our studied subjects was 54.5%, which is lower than that in developed countries and higher than that in developing countries and the general population in Iran. Moreover, the treatment rate was 62.7% and the rate of controlled hypertension was 32.5%; both of these figures are similar to those in developed countries and higher than those in the general population of Iran. This can be explained by easier access to medical checkups and antihypertensive drugs among the staff, teachers, and students at IUMS in the current study.

The wide variations in the prevalence, awareness, treatment, and control of hypertension in different communities can be explained by dissimilarities in case ascertainment procedures and in protocols for BP evaluation. Nevertheless, what all these findings reveal is that awareness, treatment, and control of hypertension still remains a major challenge the world over. In our study, although the participants were already within the health-care system, the rates of the prevalence, awareness, treatment, and control of hypertension were far from satisfactory. On the other hand, similar to developing countries, in Iran the burden of stroke, end-stage renal disease, and heart failure secondary to hypertension is growing. Consequently, policy makers and health-care providers should seek to improve programs aimed at the prevention, detection, and treatment of hypertension.

Despite the strengths of the present study, and in particular the high response rate and the use of 2 separate BP measurements, the principal limitation is that we measured BP twice at a single visit, whereas the main guidelines^28^ recommend that hypertension be defined based on the average of at least 2 or more BP readings taken at 2 or more visits after an initial screening. 

## Conclusion

 The findings of the present study revealed that the prevalence rate of hypertension among the staff, teachers, and students at IUMS was 17.5%. Of this total, the rate of awareness, treatment, and control of hypertension was 54.5%, 62.7%, and 61.9%, correspondingly. These findings show that the percentage rates of awareness, treatment, and control of hypertension among the hypertensive subjects at IUMS are unacceptable. 

## Notes:

***This paper should be cited as: ***Pakzad B, Akbari M, Baberi F. Prevalence, Awareness, Treatment, and Control of Hypertension in an Isfahan State Institution Sample. J Teh Univ Heart Ctr 2018;13(2):65-72.
